# Patient-Specific Regulatory Network Rewiring in Inflammatory Bowel Disease: How Genetic Polymorphisms Divert Incoming Signals and Contribute to Disease Pathogenesis

**DOI:** 10.1093/ibd/izaf173

**Published:** 2025-09-07

**Authors:** Balazs Bohar, John P Thomas, Yufan Liu, Johanne Brooks-Warburton, Bram Verstockt, Nick Powell, Tamas Korcsmaros, Dezso Modos

**Affiliations:** Division of Digestive Diseases, Department of Metabolism, Digestion and Reproduction, Imperial College London, London, United Kingdom; Division of Digestive Diseases, Department of Metabolism, Digestion and Reproduction, Imperial College London, London, United Kingdom; UKRI MRC Laboratory of Medical Sciences, Hammersmith Hospital Campus, London, United Kingdom; Division of Digestive Diseases, Department of Metabolism, Digestion and Reproduction, Imperial College London, London, United Kingdom; Gut Microbes and Health Programme, Quadram Institute Bioscience, Norwich Research Park, Norwich, United Kingdom; Department of Clinical, Pharmaceutical and Biological Sciences, University of Hertfordshire, Hertford, United Kingdom; Department of Gastroenterology, Lister Hospital, Stevenage, United Kingdom; Department Gastroenterology & Hepatology, University Hospitals Leuven, KU Leuven, Leuven, Belgium; Department of Chronic Diseases and Metabolism, KU Leuven, Leuven, Belgium; Division of Digestive Diseases, Department of Metabolism, Digestion and Reproduction, Imperial College London, London, United Kingdom; Division of Digestive Diseases, Department of Metabolism, Digestion and Reproduction, Imperial College London, London, United Kingdom; Gut Microbes and Health Programme, Quadram Institute Bioscience, Norwich Research Park, Norwich, United Kingdom; NIHR Imperial BRC Organoid Facility, Imperial College London, London, United Kingdom; Gut Microbes and Health Programme, Quadram Institute Bioscience, Norwich Research Park, Norwich, United Kingdom; Division of Systems Medicine, Department of Metabolism, Digestion and Reproduction, Imperial College London, London, United Kingdom

**Keywords:** Single nucleotide polymorphisms, Inflammatory Bowel Disease, Systems genomics, Gene regulatory networks, Signalling pathways, Crohn's disease, Ulcerative colitis

## Abstract

**Background:**

Intestinal cells receive incoming signals from neighboring cells and microbial communities. Upstream signaling pathways transduce these signals to reach transcription factors (TFs) that regulate gene expression. In inflammatory bowel disease (IBD), most single nucleotide polymorphisms (SNPs) are in non-coding genomic regions containing TF binding sites. These SNPs can alter TF binding affinity, leading to regulatory shifts: TFs may lose or gain binding sites, causing a significant rewiring of the incoming signals regulating gene expression. Understanding this rewiring offers critical insights into the cellular mechanisms driving IBD pathogenesis.

**Methods:**

To investigate this rewiring, we developed a systems genomics pipeline and analyzed individual genotype data from 2636 IBD patients to infer the incoming signals affecting patient-specific gene regulatory networks. Our in silico approach predicted changes in the repertoire of TFs binding to genomic loci due to IBD-associated non-coding SNPs in each patient compared to healthy controls. By functionally annotating the TFs in disease and healthy states, we highlighted the rewiring of upstream signaling pathways that may arise due to IBD-associated SNPs.

**Results:**

We revealed that diverse non-coding SNP combinations in IBD patients lead to functional switches from healthy signals to disease-associated signals, capturing patient heterogeneity while uncovering common upstream regulators driving disease pathogenesis. Notably, rewired incoming signals belonged to key functional processes such as pro-inflammatory immune responses, epithelial barrier dysfunction, stress responses, wound healing, and antimicrobial defense pathways.

**Conclusions:**

In summary, this work highlights the importance of personalized investigation of signaling processes upstream of genetic polymorphisms to gain a more comprehensive understanding of IBD pathogenesis.

Key Messages *What is already known?*

• GWAS studies have shown that most IBD-associated single-­nucleotide polymorphisms (SNPs) are located within non-coding genomic regions, but their role in IBD pathogenesis is largely unknown.

*What is new here?*

• Using a novel systems genomics pipeline to individually analyze genotype data from 2636 IBD patients, we identified how IBD-­associated non-coding SNPs alter transcription factor binding and rewire upstream signaling pathways that regulate gene expression in a patient-specific manner.

*How can this study help patient care?*

• These novel insights that integrate genetic susceptibility with extrinsic/environmental signals into the cellular signaling hierarchy in IBD offers a promising foundation for the future development of personalized therapies.

## Introduction

Inflammatory bowel disease (IBD) is an umbrella term for a group of chronic immune-mediated disorders predominantly affecting the gastrointestinal tract, of which Crohn’s disease (CD) and ulcerative colitis (UC) are the two main clinical subtypes.^1^ These disorders arise due to complex interactions between environmental factors and multiple genetic risk loci, ultimately manifesting as pathological immune activation in the gut. Patients with IBD experience significant morbidity due to symptoms such as bloody diarrhea, abdominal pain, and weight loss.^2^ Globally, the number of individuals living with IBD is on the rise, with most recent estimates forecasting prevalence rates to surpass 1% by 2030 in the United Kingdom and other Western nations.^3^ There is still no cure for IBD. In addition, despite tremendous progress with the advent of biologics and small molecule therapies over the past decade, the majority of patients fail to achieve long-lasting remission.^4^

These shortcomings highlight the need for a deeper understanding of the heterogeneous mechanisms that drive the development of IBD across the patient population. In particular, the causal mechanisms interlinking genetic risk loci with pathogenic signaling pathways remain largely elusive and need to be further characterized.^5^ These genetic risk loci encompass coding variants, non-coding variants, and structural variants (eg, insertions, deletions, inversions). Genome-wide association studies (GWAS) in IBD have been particularly successful in identifying genetic variants in the form of single nucleotide polymorphisms (SNPs) (ie, single base-pair changes in the DNA). While SNPs located in coding regions, such as those affecting the *NOD2*^6^ and *IL23R* genes,^7^ have provided important mechanistic insights into IBD pathogenesis, the vast majority of IBD-associated SNPs reside within non-coding regions of the genome which are challenging to functionally annotate.^8^ These regions contain transcription factor binding sites (TFBSs) that transcription factors (TFs) bind to regulate gene expression and influence downstream cell signaling networks.^9^ To predict the functional consequences of disease-associated non-coding SNPs on gene regulatory networks and signaling networks, we recently developed a systems genomics workflow: the integrated single nucleotide polymorphism network platform (iSNP).^10^ By using genotype data from a cohort of UC patients, we modeled how disease-associated non-coding SNPs can disrupt downstream cell signaling networks by impacting the binding of TFs to TFBSs in promoter and enhancer regions of the genome in a patient-specific manner. This enabled stratification of patients into distinct clusters based on genotype-driven pathogenic pathways, demonstrating the power of personalized systems genomics modeling in uncovering molecular heterogeneity. This work focused on the downstream effects of non-coding regulatory SNPs by harnessing the power of network biology to predict the cumulative effect of SNPs on multiple SNP-affected proteins and their first neighbor interactors in the human signaling network.

While our previous work provided novel insights into the downstream impact of non-coding SNPs in IBD, little is known about the upstream, incoming signals that influence disease-associated TFs. Activation of TFs is influenced by a variety of incoming signals triggered by environmental and intrinsic cues, such as microbes and cytokines, respectively.^11^ As disease-associated non-coding SNPs can result in the binding of different TFs in the disease state compared to the healthy state, different incoming signals may influence TF activity in patients with IBD compared to the healthy (non-IBD) population. Characterizing and understanding these incoming cues could unveil valuable insights into the lesser-known molecular drivers of IBD that via patient-specific non-coding genetic risk loci ultimately impact downstream cellular processes.

Only limited studies to date have attempted to characterize the upstream or incoming signaling events involved in IBD pathogenesis. These studies suggest that the gut microbiome may upregulate inflammatory pathways in IBD through the binding of gut bacterial peptides to host signaling proteins that modulate TF activity^12^ or through Toll-like receptor activation that triggers upstream signaling cascades.^13^ In addition to inflammation, other studies have identified upstream signaling pathways leading to intestinal permeability and wound healing in IBD.^14^ However, these investigations have not explored how incoming signals may impact genetic risk loci. More broadly, beyond IBD, few studies have attempted to connect the upstream signaling layer to GWAS candidate genes.^15^ Instead, existing research has largely focused on determining the downstream consequences of SNP-associated genes using transcriptomics, proteomics, and/or metabolomics, which measure the end points of signaling cascades.^16^

To address this gap, we present a novel in silico workflow that leverages systems genomics methods and patient-specific genotype data from a large cohort of UC and CD patients to characterize the putative incoming signals and regulatory rewiring that establishes gene regulatory networks in IBD. To the best of our knowledge, this is the first study to systematically characterize the rewiring of upstream signaling pathways that may arise in the context of disease-associated regulatory SNPs in a patient-specific manner, thereby shedding light on a poorly characterized layer of IBD pathogenesis.

## Materials and Methods

### Sources of SNP data

Immunochip data was obtained from participants in the Leuven IBD Biobank which encompassed 1695 patients with CD and 941 patients with UC. All individuals provided written consent to participate in the Leuven IBD Biobank under the study protocol approved by the institutional review board (B322201213950/S53684). The patient demographics of this cohort are summarised in Table S1.

### Modeling the impact of non-coding SNPs on transcription factor binding sites

From the patient-specific genotype data, we identified non-coding SNPs previously associated with IBD and then modeled the impact of these SNPs on TFBSs, following the protocol of Brooks-Warburton et al.^10^ Briefly, SNPs associated with IBD were first extracted from two landmark studies: Jostins et al (2012)^6^ and Farh et al (2015).^17^ Jostins et al performed a meta-analysis of 15 IBD GWAS, followed by validation using Immunochip data from an independent cohort, identifying 193 genome-wide significant independent signals (*P* < 5 × 10^−8^). Since some of these signals represented associations to the same underlying functional unit, the authors merged these signals into 163 distinct genomic loci. To refine our selection and focus on likely causal variants, we cross-referenced these GWAS hits with the fine-mapped SNPs from Farh et al (2015), who applied statistical fine-mapping based on linkage disequilibrium (LD) and functional annotations to identify candidate causal variants within each locus. In addition to SNPs that overlapped between the Jostins et al and Farh et al datasets, we also included IBD-associated fine-mapped non-coding SNPs uniquely identified by Farh et al, thereby broadening our inclusion of high-confidence candidate regulatory variants.

Next from these SNP loci, we identified those that overlapped with known regulatory regions of the genome (promoter and enhancer regions). The distribution of SNPs and TFs in promoter and enhancer regions for UC and CD patients in our cohort is summarized in Table S2. Promoters were defined as sites that are 5 kilobases (KB) upstream of the transcription start site (TSS) and downstream until the end of the first exon of the particular gene, according to the UCSC genome browser.^18^ Enhancer regions were defined based on the HEDD database (downloaded August 13, 2023),^19^ which integrates information from ENCODE,^20^ FANTOM5,^21^ and the Epigenomics Roadmap.^22^

We evaluated the potential impact of these SNPs located in regulatory genomic regions on transcription factor binding. To do this, we first obtained the TFBSs of 949 TFs from the JASPAR database (downloaded October 10, 2023).^23^ Then, we used the Regulatory Sequence Analysis Tools (RSAT) (version 2018.8.1)^24^ and Find Individual Motif Occurrences (FIMO) tool (version 5.1.1),^25^ to identify the TFs likely to bind to genomic loci containing non-coding SNPs. For further analysis, we kept TFBSs which were predicted to be perturbed either due to a gain or loss of binding site as a result of a regulatory SNP. To model how this compares to the healthy state, we also determined the TFs expected to bind to the same genomic loci in the presence of the protective (ie, non-risk) allele. Only bi-allelic SNPs were included in this analysis. Two IBD-associated non-coding SNPs in our patient cohort were found to impact the same gene regulatory region but were not collapsed into a single representative SNP. Instead, they were treated independently, in order to preserve the inherent complexity of regulatory architecture in complex diseases and to reflect the probabilistic nature of fine-mapping from Farh et al, which had already accounted for LD structure in causal inference.^17^

### Identification of transcription factor switches and gene ontology switches

**Figure 1.**
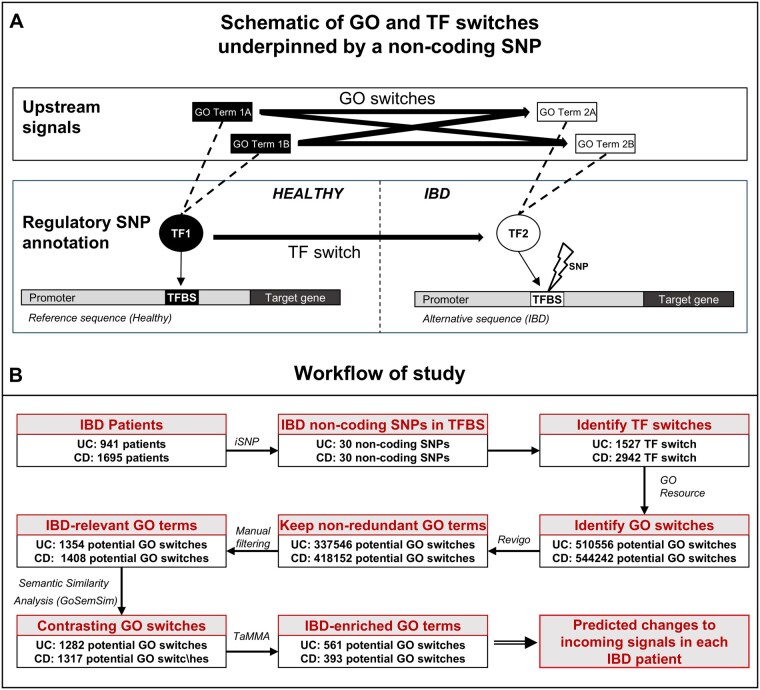
A systems genomics workflow to identify transcription factor and Gene Ontology switches from genotype data. (A) The visual representation of transcription factor (TF) and Gene Ontology (GO) switches. A non-coding SNP can result in different TFs binding to the same regulatory region of the genome, leading to distinct TFs acting in the healthy and disease states. We defined this change as TF switches. These different TFs can be associated with different biological processes between the healthy and disease states, which we defined as GO switches based on the processes we identified using the GO resource. (B) An overview of the analytical workflow. For details of each step, see the Methods.

Subsequently, for each regulatory SNP we selected, we discerned the predicted changes in the TFs associated with the healthy state and the disease state. We defined these as “TF switches” for each non-coding SNP (Figure 1). Next, we predicted the biological processes associated with TFs in the healthy state and the disease state for each regulatory SNP using the Gene Ontology resource Biological Process domain (downloaded February 22, 2024). We termed the differences in biological processes between the healthy and disease states for each regulatory SNP as “Gene Ontology switches” (GO switches).

### Shortlisting the IBD-relevant GO switches

To improve the specificity of the GO switches, we used the Revigo software (version 1.8.1)^26^ to remove redundant GO terms within the list of healthy and disease-associated GO terms. Revigo uses the SimRank algorithm to measure semantic similarity between GO terms, allowing the tool to merge similar GO terms and reduce redundancy effectively. We used Revigo with a 0.5 cutoff and the option to remove similar GO terms, with the “SimRel” semantic similarity measure.

Next, we implemented a manual filtering step to define GO switches relevant for IBD. In this step, to filter the remaining GO switches for relevant GO functions, two IBD systems medicine experts (T.K. and D.M.) independently created an IBD-relevant GO term list. These two lists were merged together resulting in 256 unique GO terms (Table S3). This step was necessary to reduce the number of GO terms from 2559 in UC and 2617 in CD to 144 and 138 respectively, allowing us to focus our analysis on incoming signals that are relevant in IBD.^27^ While this may have excluded novel GO terms that are not yet well-established in IBD, the goal of this analysis was to connect known IBD-associated signals to known IBD-associated SNPs.

Then, we used another semantic similarity-based analysis between the healthy and disease-associated GO terms to identify GO switches containing the most contrasting GO terms. To do this, the healthy and disease-associated GO terms were converted to GO IDs using the GO.db (version 3.18.0)^28^ and AnnotationDbi (version 1.64.1)^27^ packages in R. Then, semantic similarity analysis was performed between the healthy and disease-associated GO IDs for each GO switch using the GOSemSim package (version 2.28.1)^29^ in R. We used the “Relevance” semantic similarity metric with a cutoff of 0.5. Following this, GO switches that had a semantic similarity score of <0.5 were kept for further analysis, representing the most contrasting GO switches.

In the final filtering step, we kept only those GO switches that contained GO terms that were enriched among IBD patients compared to healthy controls from real-world, clinical datasets. For this, we used the TaMMA resource (downloaded 09/03/2023),^30^ which is the largest meta-analysis of bulk-transcriptomics data in IBD involving 3853 individuals with IBD from 26 independent studies. In TaMMA, these studies are analyzed using a standardized computational pipeline and batch corrected for data harmonization and simultaneous comparisons. Thus, it is a highly powered resource for identifying the likely biological processes relevant in IBD patients. Differential expression gene (DEG) lists from the colon and rectum of UC patients compared to the colon and rectum of healthy individuals were downloaded from TaMMA. Gene transcripts with a log2-fold change of >0.5 and Benjamini-Hochbberg adjusted *P* value of <0.05 were selected for functional over-­representation analysis against the background of gene transcripts present in TaMMA, using the enrichplot R package (version 1.22.0).^31^ We selected these thresholds to capture a broad range of disease-associated processes, including those with modest expression changes or those observed in only a subset of patients, while excluding processes that are unlikely to be relevant in IBD. However, we acknowledge that filtering based on population-level differential expression data may overlook rare, patient-specific pathways. GO terms with an adjusted *P* value of <0.01 were deemed to be significantly overrepresented biological functions in the UC colon and rectum compared to the healthy colon and rectum. The significant GO terms in the UC colon and UC rectum were then combined, and duplicates were removed. These steps were repeated using DEGs from the ileum, colon, and rectum of CD patients compared to healthy individuals present in TaMMA. These significant disease-associated GO terms were used to create the final, shortened list of IBD patient-relevant GO switches.

### Patient-specific network visualization of TF and GO switches

**Figure 2.**
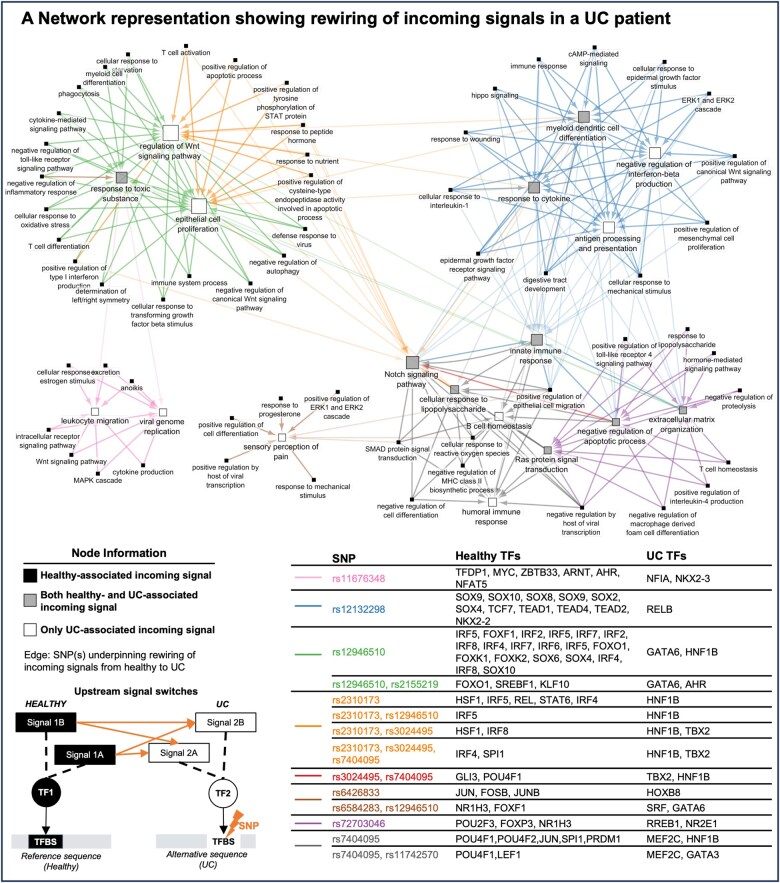

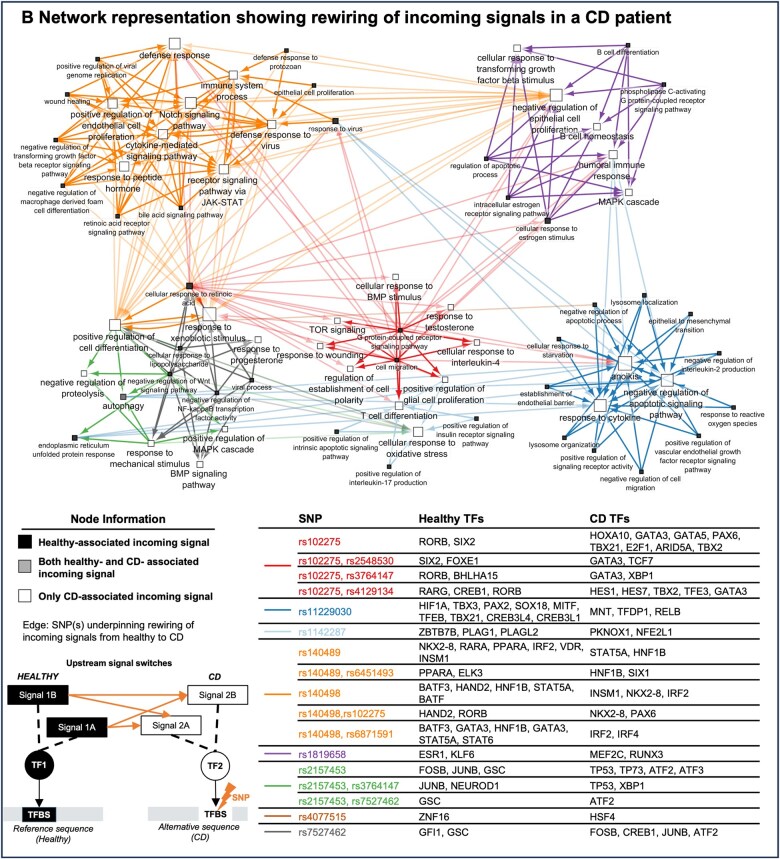
Patient-specific representative networks of functional rewiring in an (A) ulcerative colitis patient and a (B) Crohn’s disease patient. Edges represent non-coding single nucleotide polymorphism(s) underpinning the rewiring of TFs and their associated functions (ie, incoming signals acting on the genome) between the healthy and disease states. White nodes represent disease-associated incoming signals, black nodes represent healthy-associated incoming signals, while grey nodes represent incoming signals that were present in both healthy and disease states. Node size is proportional to the number of incoming signals.

We ran this workflow for each individual patient in our cohort, enabling us to determine patient-specific TF switches and GO switches. Using Cytoscape^32^ (version 3.10.1), we visualized this information, with nodes representing GO terms and edges representing non-coding SNPs underpinning switches between GO terms mediated by TF switches (Figure 2).

After running our workflow on each patient, we calculated the percentage difference in occurrence of disease-associated and healthy GO terms in our cohort of UC and CD patients. We calculated this difference between the percentage of GO switches using base R (version 4.3.2), allowing us to determine which GO terms were overall lost (percentage difference <0%) or gained (percentage difference >0%) as a result of IBD-­associated non-coding SNPs in our cohort of UC and CD patients as a whole. The GO terms with the largest percentage differences were visualized in R (version 4.3.2).

### Sensitivity analysis

The manual filtering step in our workflow reduced the number of GO switches from 337 546 to 1354 in UC and from 418 152 to 1408 in CD, making it the largest filtering step in our workflow. To test the sensitivity of the workflow to the manual filtering step, we performed a sensitivity analysis, where we omitted the manual filtering step and re-ran the workflow using only the Revigo analysis, the TaMMA enrichment analysis, and semantic similarity analysis in the workflow. When the manual filtering step was omitted, the number of GO switches was reduced only to 8608 in UC and 5127 in CD, giving rise to less IBD-specific results. When comparing the two outputs, we found that the manual filtering step excluded general GO terms such as *response to activity* and *limb morphogenesis*, or terms unrelated to IBD such as *germ cell migration* or *oocyte growth*, which would have provided limited biologically relevant insights.

## Clustering Analysis

We next evaluated whether patient heterogeneity can be captured using clustering analysis of the patient-specific GO switches and TF switches. Each patient was represented by the number of GO terms or TFs gained or lost relative to the healthy state. After scaling each feature matrix, k-means clustering was applied (scikit-learn, version 1.3.2) independently to the GO and TF matrices to identify subgroups of patients with similar switching patterns. The optimal number of clusters (k) for each feature set was determined empirically by evaluating both the silhouette score and the inertia across a range of k values to balance cluster compactness and separation. Principal component analysis (PCA) was applied to each feature matrix to visualize the distribution of clusters. We then identified the GO terms significantly enriched in specific clusters. For normally distributed features (tested using Shapiro–Wilk test), we applied one-sided *t* tests to compare values in each cluster higher than the rest. For non-normal features, we used the one-sided Mann–Whitney *U* test. *P* values were adjusted using the Benjamini–Hochberg method, and features with adjusted *P* values below 0.05 were considered significantly enriched in the corresponding cluster. The average normalized values of significantly enriched GO terms was visualized using a heatmap.

### Permutation-based statistical analysis

To evaluate the specificity of the gain and loss of GO terms in the context of IBD, we performed a two-stage permutation testing strategy.

First, we tested whether the overall number of lost and gained GO terms in IBD patients could be explained by random variation in regulatory regions, rather than disease-specific effects. From all possible SNPs present in the Single Nucleotide Polymorphism Database (dbSNP) (Build 144; downloaded May 14, 2025),^33^ we randomly selected 69 and 90 SNPs located in enhancers or promoters to match the total number of SNPs present in UC patients and CD patients in our cohort, respectively. Only SNPs with no known association with IBD were selected. This random selection was repeated 1000 times resulting in 1000 random SNP sets representing 1000 non-IBD disease states. These SNP sets were then processed through our entire pipeline, including the generation of TF switches and GO switches. This enabled us to quantify the numbers of gained and lost incoming signals under the null model. By comparing the incoming signals lost or gained in IBD patients against the distributions of gained and lost incoming signals from these random disease states, we calculated *z*-scores (Equation 1) and *P* values, using the SciPy stats method. Here, *rv* is the percentage of GO terms gained or lost in IBD patients; $\underline{\mathrm{random}\;}$ is the average gain/loss of GO terms in the random disease states; and *SD* is the standard deviation of the random permutations:


(1) $$z=\frac{rv\;-\;\underline{\mathrm{random}}}{{SD}_{\mathrm{random}}}.$$

This method tests the pipeline with a null hypothesis that there is no difference between the total number of GO terms gained or lost between IBD and non-IBD disease states. This permutation testing was undertaken both before and after the filtering steps in our pipeline outlined earlier. Together this allowed us to evaluate whether the overall network rewiring of GO terms observed in the IBD patients in our cohort is different to that expected by chance, and the extent to which the filtering steps influenced the expected null distribution of gained and lost GO terms.

Next, to identify specific GO terms that were gained or lost significantly more or less in IBD patients than expected by chance, we performed a patient-matched permutation analysis. For both UC and CD, we generated random non-IBD patient cohorts by sampling the same number of SNPs per patient as in our real cohort, out of the regulatory SNPs generated from 1000 random non-IBD disease states from our first permutation test. Then, we ran the pipeline (excluding the REVIGO semantic similarity filter due to computational limitations) to capture the expected frequency distribution for each GO term gained or lost due to random regulatory SNPs. We calculated a *z* score for each GO term similar to eqn (1), but here *rv* denotes the proportion of UC or CD patients in our IBD patient cohort for whom a GO term is gained or lost; $\underline{\mathrm{random}\;}$ is the average proportion of patients in the random non-IBD patient cohorts for whom the same GO term is gained or lost; and SD is the standard deviation of this proportion. In this way, we were able to pinpoint GO terms that were significantly rewired in IBD patients beyond what is expected by chance.

Together, these two complementary permutation approaches tested both the overall and GO term-specific rewiring of incoming signals in the context of IBD.

## Results

### Modeling patient-specific regulatory rewiring

We first identified TF switches by comparing the TFs predicted to bind to the risk and non-risk alleles at IBD-associated non-coding SNPs present in each IBD patient in our cohort. Across the entire cohort, 135 TFs were predicted to bind to SNP-affected TFBSs in UC patients, while 161 were predicted to bind to SNP-affected TFBSs in CD patients. Of these, 53 TFs were shared between both UC and CD patients (Figure S1). To assess the relevance of the identified TFs to IBD, we conducted a literature review. We focused on a total of 108 TFs that was gained in at least 1 patient with UC or CD. We found that the majority of these TFs have previously been associated with IBD. Among them, 55 TFs have functionally characterized roles, with most supported by experimental evidence and a few inferred through computational predictions (Table S6). Notably, 37 TFs (approximately one-third) have not been directly linked to IBD in the existing literature. However, many of these TFs may play plausible roles in IBD pathogenesis given that they regulate relevant pathways or biological processes. For instance, TEAD1 is a component of the Hippo pathway that has been found to exert immunomodulatory effects in murine models of colitis,^34^ and ARID5A, regulates the expression of genes downstream of the Th17/IL-17 axis,^35^^,^^36^ a pathway known to play a critical role in IBD pathogenesis.^37^

We then functionally annotated the TFs in each patient, to determine GO switches, which are the proxies for the change in upstream incoming signals (Figure 1A). With our workflow (Figure 1B), we identified 510 556 GO switches in UC patients and 544 242 GO switches in CD patients across our entire cohort. As these switches were often redundant, unrelated to IBD, or too generic, we applied a series of filtering steps (see Methods for details). The filtering steps identified 561 GO switches in UC and 393 GO switches in CD as the most relevant and contrasting switches between healthy and disease states (Table 1). Subsequent sensitivity analysis confirmed the role and order of each filtering step (see Methods).

**Table 1.** Gained and lost transcription factors (TFs) and signals (gene ontology biological processes) in UC and CD.

	Disease	Gained	Gained and lost	Lost	Switches
**TFs**	UC	59	0	76	1527
**Signals (GO terms)**		26	58	60	561
**TFs**	CD	75	0	86	2942
**Signals (GO terms)**		16	81	41	393

### Patient-specific rewiring of incoming signals

To map the putative changes of incoming signals, we visualized this rewiring with networks. This visualization illustrates how an incoming signal (GO term) in the healthy state switches to another incoming signal in the disease state, indicated by an arrow connecting the two signals (ie, forming an arrow from the healthy state-associated GO term to the disease state-associated GO term). In doing so, we highlight the functional rewiring of upstream signaling processes that are likely to occur in IBD in the context of non-coding SNPs. Using our analytical pipeline, we created these rewiring networks for each individual patient, allowing us to understand patient-specific differences. Representative example networks from a UC patient and a CD patient from the analyzed cohort are depicted in Figure 2A and 2B, respectively.

As shown in these examples, certain incoming signals were relevant in both healthy and disease states by acting through different TFs. This indicates that the same incoming signal may be rewired to act on different target genes in the disease state compared to health due to non-coding SNPs. Our network visualization also revealed that non-coding SNPs in IBD patients may rewire multiple healthy state-associated signaling pathways into fewer, disease-associated key signals.

### Cohort analysis reveals a significant rewiring of incoming signals in IBD

To gain a better understanding of the overarching rewiring of incoming biological signals occurring in IBD, we analyzed the patient-specific findings across our entire cohort of UC and CD patients. Then we calculated the overall difference in percentage occurrence of GO terms between the healthy and disease states in our cohort.

**Figure 3.**
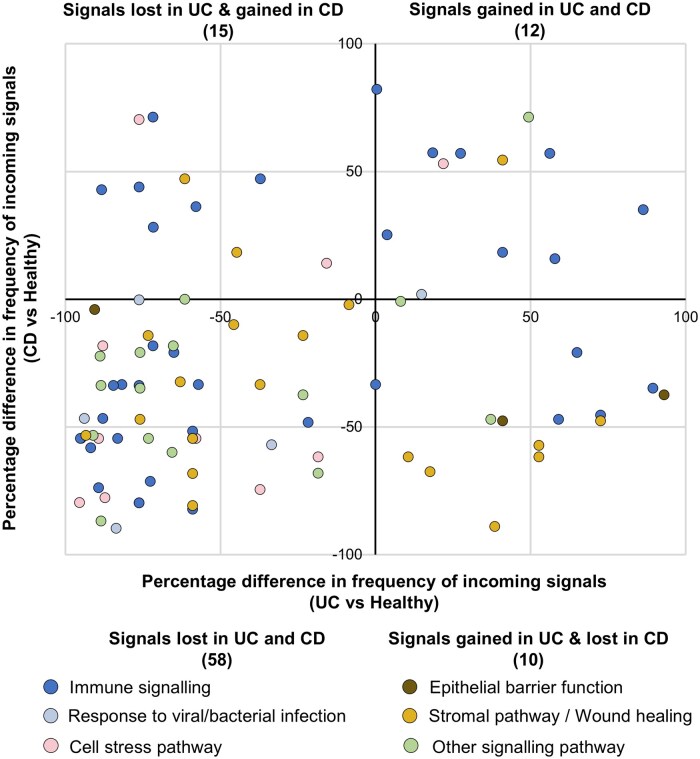
Frequency of shared incoming signals to TF binding sites in UC and CD patients within the study cohort. The x-axis shows the percentage difference in the frequency of GO term occurrences in UC patients relative to the healthy state, while the y-axis displays the same percentage difference for CD patients compared to the healthy state. Note that processes unique to UC or CD are not shown here.

This analysis revealed that 144 processes and 138 processes were gained or lost in UC patients and CD patients, respectively, compared to the healthy state. Ninety-five of these incoming biological signals were common to UC and CD, indicating a significant overlap in the upstream signaling mechanisms underpinning both types of IBD (Figure 3). In both UC and CD, the lost or gained signals could be grouped into 6 categories: (1) immune signaling, (2) response to viral or bacterial stimulus, (3) cellular stress, (4) epithelial signals, (5) stromal or wound healing, and (6) other signaling pathways (Figure 3). We found that diverse regulatory SNPs contributed to the rewiring of these incoming signals, although certain SNPs underpinned more signal rewiring than others (Table S4 for UC and Table S5 for CD). This indicates pleiotropic mechanisms underlying the disruption of upstream, incoming signals in IBD, with certain variants disproportionately driving the loss or gain of upstream signaling pathways irrespective of their cohort frequency.

In both UC and CD, we observed that far more incoming signals were lost than gained compared to the healthy state (left lower quadrant, Figure 3). To assess the specificity of these findings to IBD, we performed permutation testing of our pipeline by generating null distributions of gained and lost incoming signals from 1000 random non-IBD disease states, each represented by a set of random non-coding regulatory SNPs not associated with IBD. These distributions revealed that the greater loss of incoming signals compared to gained signals was observed both in IBD and random non-IBD disease states after the filtering steps of our pipeline (Figure S2 A-D), indicating that the consolidation of incoming signals is independent of regulatory SNP selection. However, we found that after filtering, incoming signals were gained more frequently in IBD patients than expected by chance and lost less frequently than expected by chance (*P* < 0.001, *z* score statistical test, Figure S2E-H), likely reflecting the IBD specificity of the GO terms used in the filtering steps.

**Figure 4.**
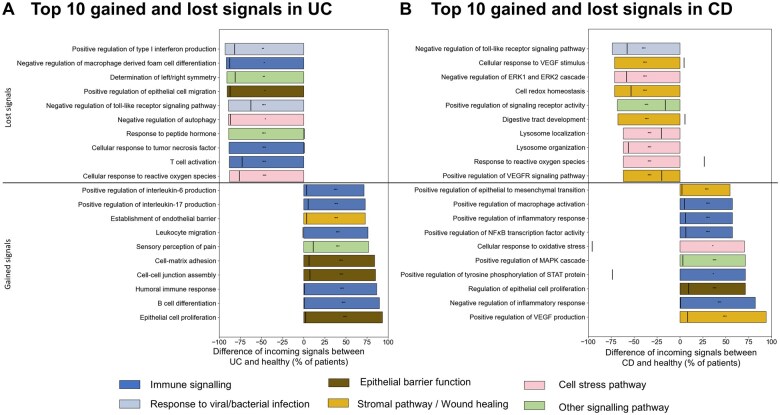
Most frequent upstream incoming signals that rewire the gene regulatory network in UC (A, B) and CD (C, D) patients. (A) Top 10 significant lost and gained signals in UC patients. The black line is the expected value from permutation testing. (B) Top 10 significant lost and gained signals in CD patients. The black line is the expected value from permutation testing. Statistics: Benjamini-Hochberg corrected *z* tests compared with perturbed GO terms. For the remaining lost and gained incoming signals see Figure S3.

To identify the GO terms that were significantly rewired in IBD patients, we performed a patient-matched permutation analysis. We focused on GO terms that were lost or gained significantly more frequently in UC and CD patients than expected by chance. There were greater numbers of such statistically significant lost signals than gained. The top 10 most frequently gained and lost significantly incoming signals for UC and CD in our cohort that deviated in this way from the null distribution are depicted in Figure 4A and 4B, respectively.

These most frequently gained and lost signals related to immune signaling, highlighting extensive rewiring of regulatory mechanisms associated with the immune system, particularly cytokine signaling and both innate and adaptive immune cell activation in IBD. Incoming signals related to host responses against bacteria or viruses were also frequently lost in both diseases (eg, negative regulation of toll-like receptor signaling), indicating impaired regulation of host mechanisms against pathogenic stimuli. The multiple mesenchymal and epithelial cell processes that were affected in both CD and UC suggest a pathological rewiring of gut barrier regeneration and maintenance, which could predispose IBD patients to a “leaky gut.”^38^ Signals related to cellular stress, including response to reactive oxygen species and autophagy, were also frequently rewired in both UC and CD due to a disease-associated dysregulation indicated by loss of multiple incoming signals.

While IBD patients had various underlying genotypes, at the level of incoming signal changes, we identified signals that were almost ubiquitously lost in all patients. For example, 93% of the UC patients in our cohort lost the signal related to the positive regulation of type 1 interferon production—a pathway critical to the frontline defense against viral infection (Figure 4A). In the top 10 gained signals in UC patients, we noted multiple immune system and epithelial barrier function–related signals. This indicates a genetic predisposition in which disease-associated signals result in immune dysregulation and epithelial barrier dysfunction, features characteristic of UC.

In CD, the most frequently lost signals related to cell stress responses and autophagy (eg, lysosome localization and lysosome organization), which was also observed in UC. However, compared to UC, there was a higher proportion of wound healing and mesenchymal signaling pathways that were rewired in CD patients, particularly centered around vascular endothelial growth factor (VEGF) signaling. These incoming signal changes reveal a general mesenchymal dysregulation in CD patients, predisposed by genetics but driven by incoming signals.

### Incoming signals stratify IBD patients to capture molecular heterogeneity

**Figure 5.**
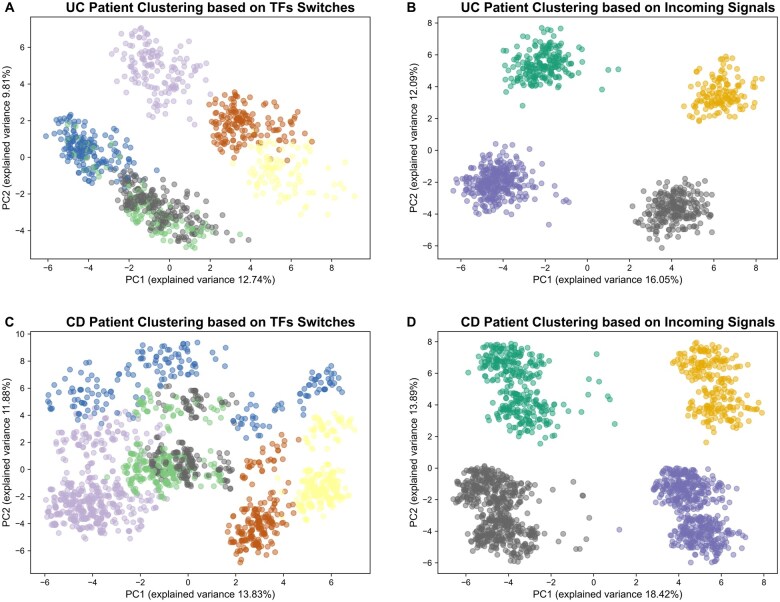
Principal Component Analysis (PCA) visualization of patient clusters: (A) UC patient clustering based on TFs switches; (B) UC patient clustering based on incoming signals; (C) CD patient clustering based on TFs switches; (D) CD patient clustering based on incoming signals.

Next, we investigated whether the patient-specific rewiring of TFs and incoming signals could dissect patient heterogeneity in our cohort. We found that in both UC and CD, clustering based on the rewiring of incoming signals, rather than TFs, more effectively captured patient heterogeneity by stratifying patients into four distinct subgroups. This was supported by higher silhouette scores for incoming signal-based clustering (Figure S4) and clearer separation of clusters in principal component analysis (PCA) visualization compared to TF-based clustering (Figure 5). We then identified the most distinct incoming signals that were gained between patient clusters and visualized these as a heatmap (Figure S5). This revealed co-occurring gained incoming signals that are likely to represent distinct molecular subgroups within UC and CD patients in our cohort.

## Discussion

We developed a systems genomics workflow to functionally annotate the reassortment of transcription factors (TFs) between healthy and disease states driven by non-coding SNPs, uncovering the rewiring of incoming signals shaping gene regulatory networks in IBD. This approach revealed a previously underappreciated layer of the cellular signaling hierarchy involved in IBD pathogenesis.

Recent insights on the role of non-coding SNPs in IBD pathogenesis indicate that they disrupt cis-regulatory gene networks by impacting TF binding sites in promoter and enhancer regions of the genome.^10^^,^^39–41^ This can result in changes to the repertoire and activity of TFs that influence gene regulation and ultimately gene expression between healthy and disease states.^42^ Characterizing the downstream impact of these gene regulatory changes due to non-coding SNPs has emerged as a major focus of research efforts in IBD and other complex diseases in recent years. This has been facilitated by the advent of state-of-the-art bioinformatics tools and experimental approaches (eg, massively parallel reporter assays and Clustered Regularly Interspaced Short Palindromic Repeats (CRISPR) screens).^43^^,^^44^ However, few studies have attempted to discern how biological signals acting upstream of disease-associated genomic loci differ in IBD compared to the healthy state. This is largely because downstream changes in cellular signaling and gene expression due to genomic variants are more readily measurable and tangible to delineate than upstream signaling pathways.^16^

**Figure 6.**
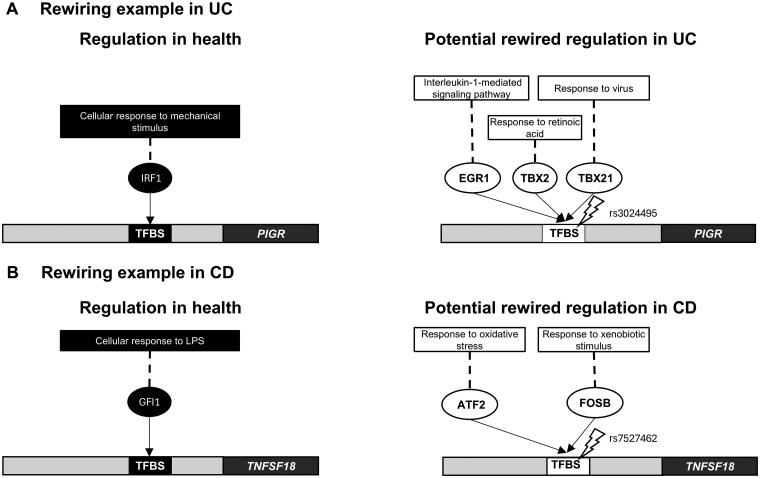
Example regulatory rewiring in UC (A) and CD (B). Based on manual curation of the literature and regulatory databases (TFLink and DoRothEA), we selected a known regulatory interaction that could become rewired due to an IBD-associated SNP.

Using the presented novel workflow, we first performed a patient-specific analysis of genotype data from a large cohort of IBD patients totaling 2636 individuals with UC or CD. This rewiring analysis revealed putative mechanisms that may contribute to IBD pathogenesis at the upstream signaling layer. To show how non-coding IBD-associated SNPs can alter the regulation of incoming signals of specific target genes, we present illustrative examples in UC and CD. In UC, we found that the regulation of the *PIGR* (polymeric immunoglobulin receptor) gene could be altered due to the SNP rs3024495 (Figure 6A). *PIGR* is involved in immunoglobulin A sensing that is dysregulated in UC.^45^ In the healthy state, IRF1 is a TF that regulates *PIGR.*^46^ However, an IBD-associated SNP (rs3024495) makes IRF1 binding less probable, while increasing the binding affinity of other TFs such as EGR1, TBX2, and TBX21 (TBET). The absence of IRF1 binding to regulate *PIGR*, coupled with the aberrant binding of other TFs to this locus, may contribute to the transcriptional dysregulation observed in IBD. Interestingly, based on the upstream signals modulating these TFs, the rewired transcriptional regulation of *PIGR* may be driven by pro-inflammatory interleukin signaling via EGR1,^47^ retinoic acid response related to cell cycle regulation via TBX2,^48^ and viral response via TBET.^49^^,^^50^ All these upstream signals are known to be relevant in IBD.

In CD, the non-coding SNP rs7527462 was found to alter the regulation of *TNFSF18*, a TNF superfamily ligand that functions as an immune costimulatory molecule^51^^,^^52^ (Figure 6B). Under healthy conditions, our analysis indicates that *TNFSF18* is regulated by GFI1, a transcriptional repressor that can be activated by lipopolysaccharide (LPS). While GFI1 is known to indirectly repress TNF and other TNF superfamily ligands by antagonising NF𝛋B activity^53^ in response to LPS in innate immune cells,^54^ our analysis suggests a more direct regulatory role for GFI1 in limiting *TNFSF18* expression. In CD, we find that rs7527462 can result in the preferential binding of alternative TFs at this locus including ATF2 and FOSB that can be activated by oxidative stress and xenobiotic stimuli, respectively. These upstream signals are known activators of these TFs.^55^^,^^56^ Notably, FOSB is a component of the AP-1 transcriptional complex, which has previously been shown to upregulate *TNFSF18*.^57^ Collectively, this example illustrates how a non-coding SNP can disrupt known tolerogenic immune regulatory mechanisms, allowing upstream environmental cues to enhance the expression of an immunomodulatory molecule that could potentially contribute to immune dysregulation in CD.

Using network rewiring analysis and patient-specific visualization (Figure 2), we revealed that various combinations of regulatory SNPs within an individual IBD patient can lead to the same outcome (ie, a switch from multiple healthy state-associated incoming signals to fewer disease-associated signals). To systematically quantify and assess the phenomena of incoming signal rewiring in IBD, we aggregated patient-specific findings across the entire UC and CD cohorts separately. This revealed a greater loss of incoming signals acting on the genome in both UC and CD, suggesting a rewiring of upstream signaling networks towards fewer IBD-associated pathways compared to the healthy state. While permutation testing revealed that this consolidation of incoming signals is independent of regulatory SNP selection (Figure S2), we identified multiple incoming signals that were lost or gained more frequently in IBD patients than expected by chance, with such statistically significant lost signals outnumbering gained signals (Figure S3).

At the same time, this analysis also unraveled nearly ubiquitous disease-associated signals whose appearance as upstream regulators can contribute to the disease pathogenesis (Figure 4). For instance, several pathognomonic immune pathways associated with IBD could be rewired in the majority of UC and CD patients in our cohort due to non-coding SNPs. In UC patients, TFs involved in the positive regulation of IL-17 and IL-6 production, leukocyte migration, and B cell differentiation were predicted to gain genomic binding sites due to disease-associated regulatory SNPs. Meanwhile in CD, regulatory SNPs were predicted to result in the binding of TFs functionally associated with the positive regulation of several critical pro-inflammatory pathways including tyrosine phosphorylation of STAT protein, macrophage activation and NF𝛋B signaling activity. Concomitantly, TFs associated with the negative regulation of key immune pathways such as macrophage activation and MHC class II biosynthesis in UC and IL-2 production and receptor signaling via JAK-STAT in CD were predicted to lose their binding sites compared to healthy individuals due to IBD-associated SNPs (Figure S3). While most of these dysregulated immune pathways are already known to be pathognomonic in IBD,^58^ our findings reveal that the intestinal mucosa of IBD patients may be genetically poised to favor pro-inflammatory signaling cascades even at the proximal end of the cellular signaling hierarchy.

In both UC and CD patients, some of the most common upstream, incoming signals predicted to be lost due to regulatory SNPs included those relating to various cell stress response pathways such as autophagy, and response to reactive oxygen species. While specific coding variants disrupting autophagy genes (eg, *ATG16L1* and *NOD2*) have been well-characterized in CD,^59^ these findings reveal a more extensive cellular dysregulation of cell stress response pathways in both UC and CD patients at the upstream signaling layer. Additionally, pathways related to epithelial barrier function such as epithelial cell proliferation were among the most frequently gained biological processes affected by non-coding SNPs in both UC and CD patients. Since epithelial barrier integrity depends on tightly regulated processes such as stem cell renewal, proliferation, differentiation, migration, and cell death, this finding highlights a genetic basis for disrupting this balance, potentially driving epithelial barrier dysfunction and excessive proliferation observed in IBD.^60^^,^^61^ Together, the upstream rewiring of incoming signals relating to immune pathways, cellular stress responses, and/or epithelial barrier function may ultimately manifest in an intestinal mucosa that is genetically predisposed to develop overactive inflammatory responses to cellular insults.

Interestingly, our analysis revealed that incoming signals related to host response to viral infection were some of the most frequently rewired in both UC and CD patients, indicating an extrinsic cellular insult that patients may be more susceptible to generating pathological responses against. For instance, negative regulation of viral genome replication was frequently gained in CD patients (Figure S3), while positive regulation of type 1 interferon production was one of the most frequently lost signals in UC patients (Figure 4A). Additionally, negative regulation of TLR signaling was frequently lost in both UC and CD patients (Figure 4A and 4B). There is increasing interest in the role of viruses in IBD pathogenesis, with some reports suggesting that gut virome dysbiosis characterized by an expansion of *Caudovirales* may drive gastrointestinal inflammation and epithelial barrier dysfunction.^62^ It remains unclear, however, whether viral dysbiosis is a cause or consequence of gastrointestinal inflammation. Our findings indicate that the normal regulation of protective host mechanisms against viral infection may be frequently lost in the context of IBD-associated non-coding SNPs. Thus, the genetic background in IBD may perturb the crosstalk that normally exists between the gut virome and the host, leading to the emergence of gut virome dysbiosis. Alternatively, incoming cellular signals from established gut virome dysbiosis may be accentuated in the host due to the genotype-driven loss of homeostatic regulatory mechanisms, resulting in pro-inflammatory responses. Further work will be required to dissect the precise role of gut virome dysbiosis in IBD pathogenesis, but our findings suggest that host genetic mechanisms may be intimately linked with this phenomenon.

Pathways associated with wound healing and the stromal compartment were also frequently rewired in our analyses in both forms of IBD, especially in CD. In CD patients, signals relating to the VEGF pathway were among the most frequently gained and lost. VEGF has been previously identified as an important mediator of intestinal angiogenesis and inflammation in IBD.^63^ In both UC and CD patients, a substantial proportion of patients gained positive regulation of epithelial to mesenchymal transition (Figure 4B and Figure S3). Epithelial-mesenchymal transition is a key wound healing pathway, and dysregulation of this process has been associated with the development of intestinal fibrosis and epithelial barrier dysfunction in IBD.^64^ In UC patients, pathways relating to the positive regulation of endothelial cell and fibroblast proliferation were frequently gained, while extracellular matrix organization was a major lost signal (Figure S3). In recent years, there has been increasing recognition that pathways involving stromal cells and the extracellular matrix are key players in IBD pathogenesis,^65^ even offering prognostic relevance relating to therapeutic response.^66^^,^^67^ These findings indicate that there may be a previously unrecognized genetic basis for dysregulated stromal pathomechanisms in IBD, which requires further study.

We next evaluated whether our patient-specific rewiring analysis could dissect patient heterogeneity in our cohort. We found that in both UC and CD, clustering based on the rewiring of incoming signals, as opposed to TFs, more effectively stratified patients into distinct subgroups (Figure 5). Although the clustering analysis revealed four clear molecular subgroups comprising co-occurring incoming signals in both UC and CD (Figure S5), as we lacked clinical metadata (such as IBD phenotypes and therapeutic response), we were unable to determine whether these molecular subgroups correspond to clinically meaningful phenotypes. As large-scale IBD biorepositories comprising genotype data and rich clinical metadata from patients emerge, future work will enable validation of these molecular subgroups in relation to clinical outcomes. From a translational perspective, this systems genomics framework offers a promising avenue for innovative precision medicine approaches aimed at targeting upstream signaling pathways. Based on our findings, we hypothesize that reversing the observed lost regulatory flexibility driven by the loss and gain of upstream signals associated with regulatory SNPs may have broad therapeutic potential as they represent proximal regulators of key pathogenic cascades in IBD. This may be achieved not only through drugs targeting specific signaling molecules but also through modulation of environmental exposures (eg, viral and bacterial triggers or xenobiotics) that feed into dysregulated signaling pathways.

While our systems genomics workflow yielded plausible and biologically relevant predictions, we acknowledge that there were limitations with our approach. Our method predicts TF binding using two complementary sequence-based approaches (RSAT^24^ and FIMO^25^). Such methods predict the strength of TF binding but can result in false-positives and do not take into account chromatin accessibility and epigenetic modifications that may be altered in IBD patients, which are intrinsic limitations of this approach.^68^ Moreover, we note that benchmarking studies have shown that motif-based predictions alone may not reliably reflect in vivo TF binding, and experimental validation with ChIP-seq or reporter assays would be required to confirm these predictions.^69^ Furthermore, although we used the GO database in our workflow, as it is the most comprehensive annotation resource of proteins,^70^ it may contain erroneous annotations. We opted not to filter the GO annotation by confidence level to increase the coverage of GO biological processes, particularly as human TFs are well-annotated compared to other organisms.^71^ Furthermore, we acknowledge that not all GO terms associated with a TF may represent incoming signals converging on genomic loci, as these may also capture downstream pathways associated with a TF. We note that by including a manual filtering step to improve the IBD-specificity of the GO analysis, we may have introduced selection bias. However, two signaling experts independently performed this process, in an attempt to reduce this bias while increasing the IBD specificity of the workflow as demonstrated in our sensitivity analysis. Thus, while this step inevitably and intentionally led to IBD-specific results, the observed rewiring of these incoming signals and how they correspond to expected IBD phenotypes were not biased by the methods. Finally, we acknowledge that our in silico workflow necessitates experimental validation, although experimental methods to dissect upstream biological pathways influencing gene regulatory mechanisms are currently limited and technically challenging.

In summary, by employing a novel systems genomics pipeline with genotype data from a large patient cohort, we gained insights into how IBD-associated genotypes can rewire the incoming biological signals acting on gene regulatory networks. This patient-specific rewiring impacts pathways relating to immune signaling, epithelial barrier function, stress response mechanisms, wound healing processes, and host response to infection. Such rewiring may render the intestinal mucosa genetically poised to favor pro-inflammatory responses, even at the proximal end of the cellular signaling hierarchy. These findings underscore the importance of investigating signaling processes upstream of genetic polymorphisms to gain a more comprehensive understanding of IBD pathogenesis.

## Supplementary Material

izaf173_Supplementary_Data

## Data Availability

To model the impact of non-coding SNPs on transcription factor binding sites, we used the Integrated Single Nucleotide Polymorphism Network platform (iSNP) that we developed earlier and is available here: https://github.com/korcsmarosgroup/iSNP. The source code for our further analyses (identification of transcription factor switches and GO switches; filtering steps; determination the most frequent go terms that were lost or gained in overall patient cohort due to noncoding SNPs; sensitivity analyses) can be found on the following GitHub link: https://github.com/korcsmarosgroup/system_genomics_analysis. The final GO switches and transcription factor switches for both UC and CD are presented in Table S7.
