# Supplementary material for: Patient-Specific Regulatory Network Rewiring in Inflammatory Bowel Disease: How Genetic Polymorphisms Divert Incoming Signals and Contribute to Disease Pathogenesis
Source: Inflamm Bowel Dis. 2025 Sep 7;31(10):2665–80. doi: 10.1093/ibd/izaf173 (PMC12558586; doi:10.1093/ibd/izaf173)
Supplement: izaf173_Supplementary_Data [file izaf173_supplementary_data.zip › Supplementary Table 1.docx]

**Supplementary Table 1. Patient demographic details**

|  | Sex | | Age at diagnosis | |
| --- | --- | --- | --- | --- |
|  | Male | Female | Mean | STD |
| Ulcerative colitis | 505 | 397 | 33.78 | 14.31 |
| Crohn's disease | 704 | 991 | 27.22 | 11.82 |
