# Supplementary material for: Patient-Specific Regulatory Network Rewiring in Inflammatory Bowel Disease: How Genetic Polymorphisms Divert Incoming Signals and Contribute to Disease Pathogenesis
Source: Inflamm Bowel Dis. 2025 Sep 7;31(10):2665–80. doi: 10.1093/ibd/izaf173 (PMC12558586; doi:10.1093/ibd/izaf173)
Supplement: izaf173_Supplementary_Data [file izaf173_supplementary_data.zip › Supplementary Table 2.docx]

**Supplementary Table 2. SNP and TF locations**

|  | Disease | Enhancer | Promoter |
| --- | --- | --- | --- |
| SNPs | Ulcerative colitis | 62.5% | 37.5% |
|  | Crohn’s disease | 44.4% | 55.6% |
| TFs | Ulcerative colitis | 58.9% | 41.1% |
|  | Crohn’s disease | 31.25% | 68.75% |
