# Supplementary figures and images for: Patient-Specific Regulatory Network Rewiring in Inflammatory Bowel Disease: How Genetic Polymorphisms Divert Incoming Signals and Contribute to Disease Pathogenesis
Source: Inflamm Bowel Dis. 2025 Sep 7;31(10):2665–80. doi: 10.1093/ibd/izaf173 (PMC12558586; doi:10.1093/ibd/izaf173)

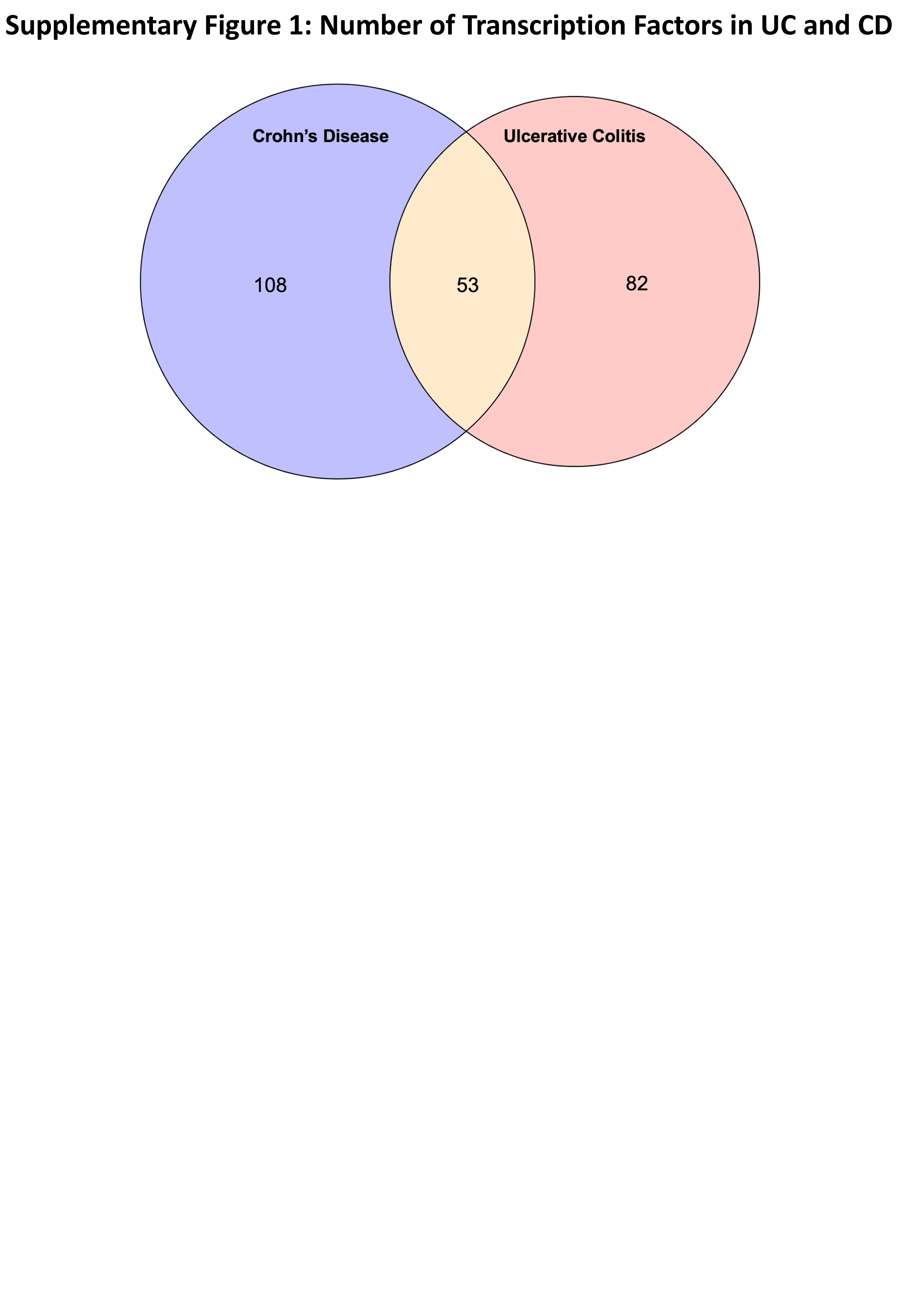

Supplement: izaf173_Supplementary_Data [file izaf173_supplementary_data.zip › Supplemenatarry_Figure1.tif]

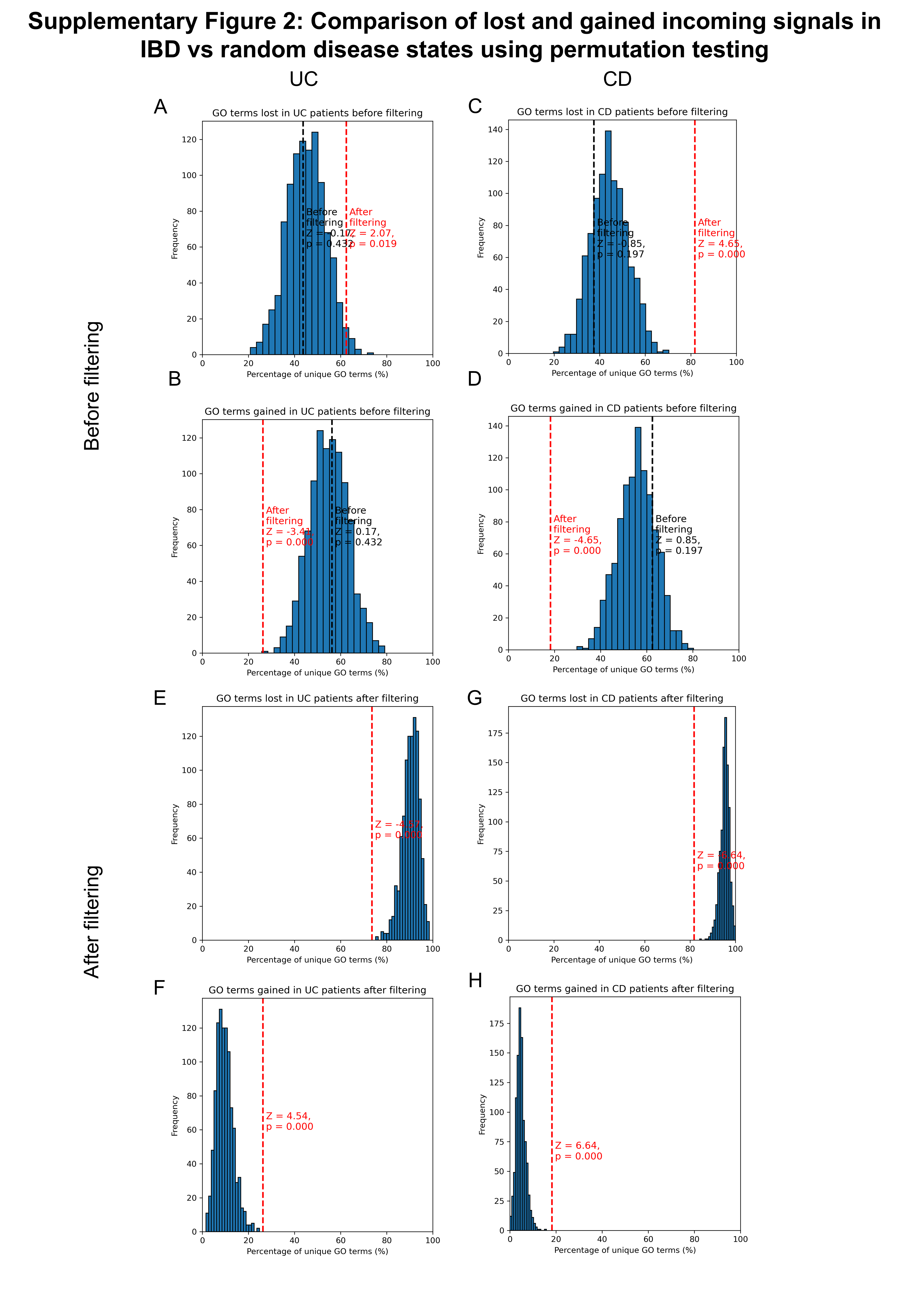

Supplement: izaf173_Supplementary_Data [file izaf173_supplementary_data.zip › Supplemenatarry_Figure2.tif]

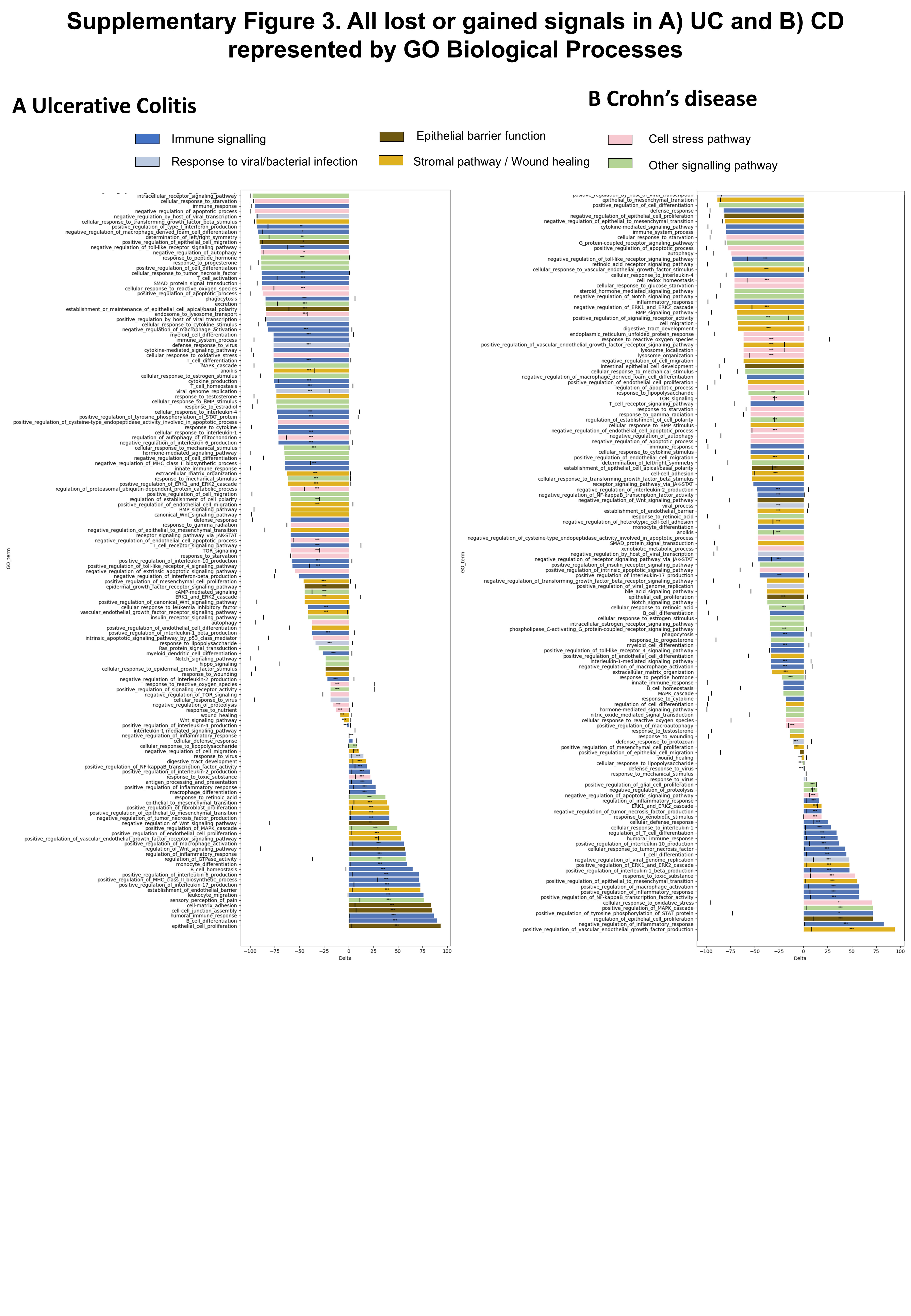

Supplement: izaf173_Supplementary_Data [file izaf173_supplementary_data.zip › Supplemenatarry_Figure3.tif]

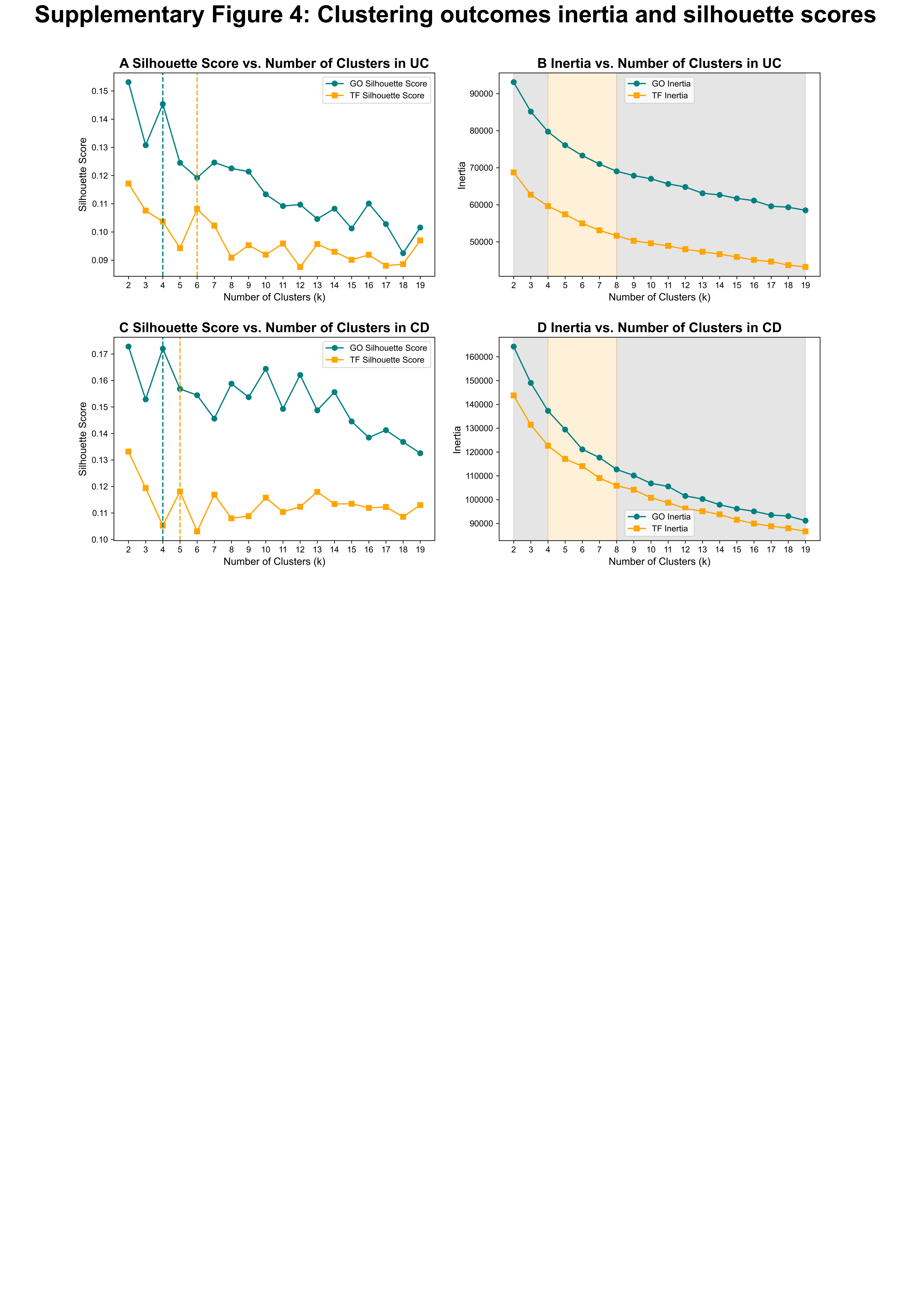

Supplement: izaf173_Supplementary_Data [file izaf173_supplementary_data.zip › Supplemenatarry_Figure4.tif]

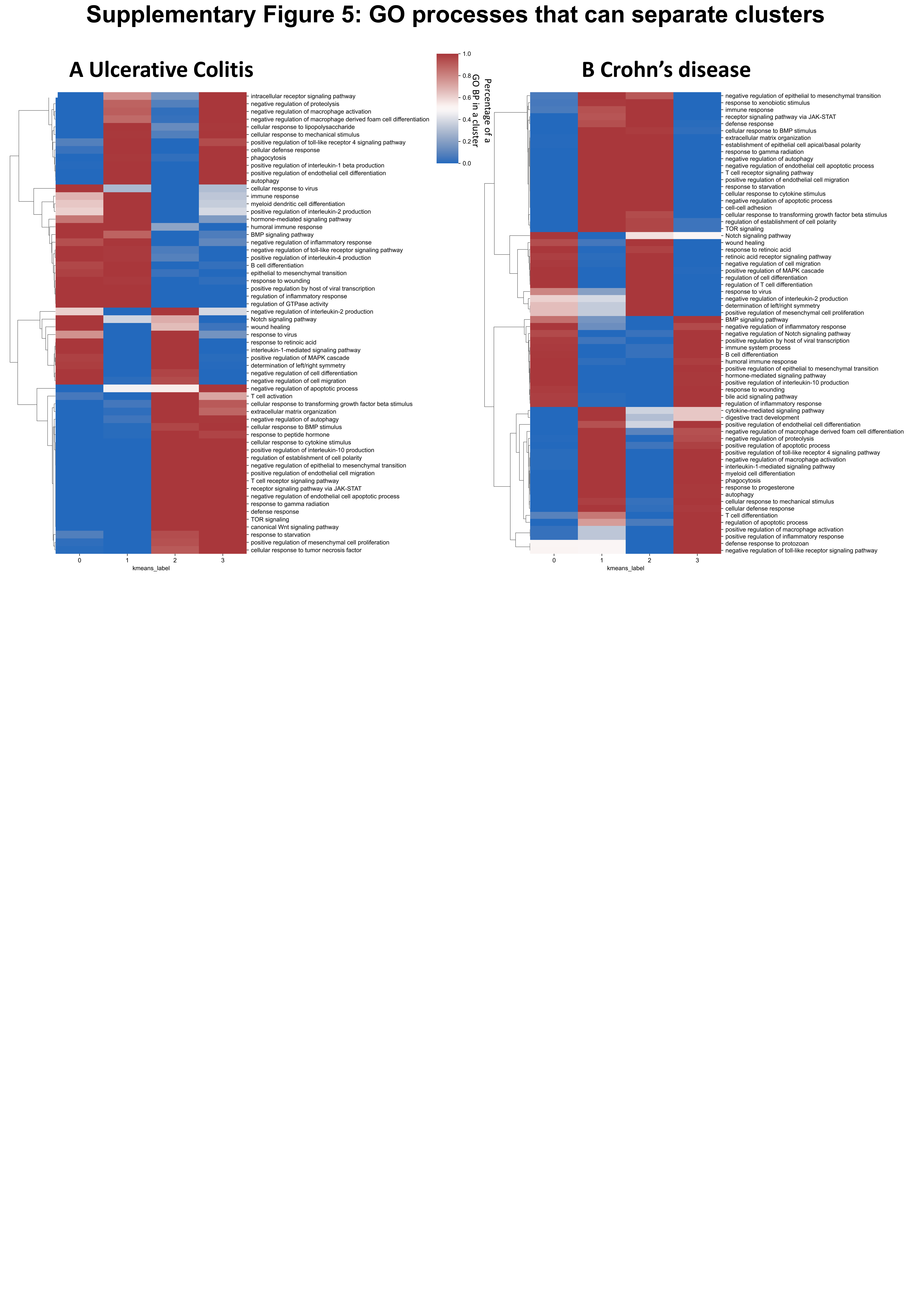

Supplement: izaf173_Supplementary_Data [file izaf173_supplementary_data.zip › Supplemenatarry_Figure5.tif]
